# Supplementary material for: Unveiling the Bioactive Potential of the Invasive Jellyfish Phyllorhiza punctata Through Integrative Transcriptomic and Proteomic Analyses
Source: Biomolecules. 2025 Aug 4;15(8):1121. doi: 10.3390/biom15081121 (PMC12383608; doi:10.3390/biom15081121)
Supplement: Supplementary file 1 [file biomolecules-15-01121-s001.zip › Suplementary Material/FileS3/PEPFOLD4/XP_065069094_12587/clusters.html]

 PEP-FOLD clusters


Download archive of all models  here   

|  |  |  |
| --- | --- | --- |
| # | file | sOPEP |
| # cluster 1 - Size: 27 |  |  |
|  | XP\_065069094\_12587-model1 | -13.1648 |
|  | XP\_065069094\_12587-model1.1 | -13.1648 |
|  | XP\_065069094\_12587-model1.2 | -13.1648 |
|  | XP\_065069094\_12587-model1.3 | -13.1648 |
|  | XP\_065069094\_12587-model1.4 | -13.1648 |
|  | XP\_065069094\_12587-model1.5 | -13.1648 |
|  | XP\_065069094\_12587-model1.6 | -13.1648 |
|  | XP\_065069094\_12587-model1.7 | -13.1648 |
|  | XP\_065069094\_12587-model1.8 | -13.1648 |
|  | XP\_065069094\_12587-model1.9 | -13.1648 |
|  | XP\_065069094\_12587-model1.10 | -13.1648 |
|  | XP\_065069094\_12587-model1.11 | -13.1648 |
|  | XP\_065069094\_12587-model1.12 | -13.1648 |
|  | XP\_065069094\_12587-model1.13 | -13.1648 |
|  | XP\_065069094\_12587-model1.14 | -13.1648 |
|  | XP\_065069094\_12587-model1.15 | -13.1648 |
|  | XP\_065069094\_12587-model1.16 | -13.1648 |
|  | XP\_065069094\_12587-model1.17 | -13.1648 |
|  | XP\_065069094\_12587-model1.18 | -13.1648 |
|  | XP\_065069094\_12587-model1.19 | -13.1648 |
|  | XP\_065069094\_12587-model1.20 | -13.0635 |
|  | XP\_065069094\_12587-model1.21 | -13.0635 |
|  | XP\_065069094\_12587-model1.22 | -13.0635 |
|  | XP\_065069094\_12587-model1.23 | -13.0486 |
|  | XP\_065069094\_12587-model1.24 | -13.0486 |
|  | XP\_065069094\_12587-model1.25 | -13.0486 |
|  | XP\_065069094\_12587-model1.26 | -13.0486 |
| # cluster 2 - Size: 15 |  |  |
|  | XP\_065069094\_12587-model2 | -13.1593 |
|  | XP\_065069094\_12587-model2.1 | -13.1593 |
|  | XP\_065069094\_12587-model2.2 | -13.1593 |
|  | XP\_065069094\_12587-model2.3 | -13.0892 |
|  | XP\_065069094\_12587-model2.4 | -13.0892 |
|  | XP\_065069094\_12587-model2.5 | -13.0892 |
|  | XP\_065069094\_12587-model2.6 | -13.0892 |
|  | XP\_065069094\_12587-model2.7 | -13.0892 |
|  | XP\_065069094\_12587-model2.8 | -13.0758 |
|  | XP\_065069094\_12587-model2.9 | -13.0758 |
|  | XP\_065069094\_12587-model2.10 | -13.0758 |
|  | XP\_065069094\_12587-model2.11 | -13.0758 |
|  | XP\_065069094\_12587-model2.12 | -13.0738 |
|  | XP\_065069094\_12587-model2.13 | -13.0738 |
|  | XP\_065069094\_12587-model2.14 | -13.0738 |
| # cluster 3 - Size: 33 |  |  |
|  | XP\_065069094\_12587-model3 | -13.1428 |
|  | XP\_065069094\_12587-model3.1 | -13.1428 |
|  | XP\_065069094\_12587-model3.2 | -13.1428 |
|  | XP\_065069094\_12587-model3.3 | -13.1428 |
|  | XP\_065069094\_12587-model3.4 | -13.1428 |
|  | XP\_065069094\_12587-model3.5 | -13.1428 |
|  | XP\_065069094\_12587-model3.6 | -13.1428 |
|  | XP\_065069094\_12587-model3.7 | -13.1428 |
|  | XP\_065069094\_12587-model3.8 | -13.1428 |
|  | XP\_065069094\_12587-model3.9 | -13.1428 |
|  | XP\_065069094\_12587-model3.10 | -13.1428 |
|  | XP\_065069094\_12587-model3.11 | -13.1428 |
|  | XP\_065069094\_12587-model3.12 | -13.1428 |
|  | XP\_065069094\_12587-model3.13 | -13.1428 |
|  | XP\_065069094\_12587-model3.14 | -13.1428 |
|  | XP\_065069094\_12587-model3.15 | -13.1428 |
|  | XP\_065069094\_12587-model3.16 | -13.1428 |
|  | XP\_065069094\_12587-model3.17 | -13.1428 |
|  | XP\_065069094\_12587-model3.18 | -13.1428 |
|  | XP\_065069094\_12587-model3.19 | -13.1428 |
|  | XP\_065069094\_12587-model3.20 | -13.1428 |
|  | XP\_065069094\_12587-model3.21 | -13.1428 |
|  | XP\_065069094\_12587-model3.22 | -13.1428 |
|  | XP\_065069094\_12587-model3.23 | -13.1428 |
|  | XP\_065069094\_12587-model3.24 | -13.1428 |
|  | XP\_065069094\_12587-model3.25 | -13.1428 |
|  | XP\_065069094\_12587-model3.26 | -13.1428 |
|  | XP\_065069094\_12587-model3.27 | -13.1428 |
|  | XP\_065069094\_12587-model3.28 | -13.1428 |
|  | XP\_065069094\_12587-model3.29 | -13.1428 |
|  | XP\_065069094\_12587-model3.30 | -13.1428 |
|  | XP\_065069094\_12587-model3.31 | -13.1428 |
|  | XP\_065069094\_12587-model3.32 | -13.1428 |
| # cluster 4 - Size: 11 |  |  |
|  | XP\_065069094\_12587-model4 | -13.1139 |
|  | XP\_065069094\_12587-model4.1 | -13.1139 |
|  | XP\_065069094\_12587-model4.2 | -13.1139 |
|  | XP\_065069094\_12587-model4.3 | -13.1139 |
|  | XP\_065069094\_12587-model4.4 | -13.1055 |
|  | XP\_065069094\_12587-model4.5 | -13.1055 |
|  | XP\_065069094\_12587-model4.6 | -13.1055 |
|  | XP\_065069094\_12587-model4.7 | -13.1055 |
|  | XP\_065069094\_12587-model4.8 | -13.0795 |
|  | XP\_065069094\_12587-model4.9 | -13.0795 |
|  | XP\_065069094\_12587-model4.10 | -13.059 |
| # cluster 5 - Size: 5 |  |  |
|  | XP\_065069094\_12587-model5 | -13.0768 |
|  | XP\_065069094\_12587-model5.1 | -13.0768 |
|  | XP\_065069094\_12587-model5.2 | -13.0768 |
|  | XP\_065069094\_12587-model5.3 | -13.0768 |
|  | XP\_065069094\_12587-model5.4 | -13.0524 |
| # cluster 6 - Size: 3 |  |  |
|  | XP\_065069094\_12587-model6 | -13.0497 |
|  | XP\_065069094\_12587-model6.1 | -13.0497 |
|  | XP\_065069094\_12587-model6.2 | -13.0497 |
| # cluster 7 - Size: 6 |  |  |
|  | XP\_065069094\_12587-model7 | -13.0488 |
|  | XP\_065069094\_12587-model7.1 | -13.0488 |
|  | XP\_065069094\_12587-model7.2 | -13.0463 |
|  | XP\_065069094\_12587-model7.3 | -13.0463 |
|  | XP\_065069094\_12587-model7.4 | -13.0463 |
|  | XP\_065069094\_12587-model7.5 | -13.0463 |
|  |  |  |
